# 3D revisualization: a new method to revisit segmented data

**DOI:** 10.1098/rsos.240375

**Published:** 2024-06-12

**Authors:** Yuzhi Hu, Ajay Limaye, Jing Lu

**Affiliations:** ^1^ CT Lab, Department of Materials Physics, Research School of Physics, Australian National University, Canberra, ACT 2601, Australia; ^2^ National Computational Infrastructure, Building 143, Corner of Ward Road and Garran 7 Road, Ward Rd, Canberra, ACT 2601, Australia; ^3^ Key Laboratory of Vertebrate Evolution & Human Origins of China, Institute of Vertebrate Paleontology and Paleoanthropology, Chinese Academy of Sciences, Beijing 100044, People’s Republic of China; ^4^ CAS Center for Excellence in Life & Paleoenvironment, Chinese Academy of Science, Beijing 100044, People’s Republic of China

**Keywords:** 3D visualization, 3D segmentation, *Drishti*, interoperability

## Abstract

3D visualization and segmentation are increasingly widely used in physical, biological and medical science, facilitating advanced investigative methodologies. However, the integration and reproduction of segmented volumes or results across the spectrum of mainstream 3D visualization platforms remain hindered by compatibility constraints. These barriers not only challenge the replication of findings but also obstruct the process of cross-validating the accuracy of 3D visualization outputs. To address this gap, we developed an innovative revisualization method implemented within the open-source framework of *Drishti*, a 3D visualization software. Leveraging four animal samples alongside three mainstream 3D visualization platforms as case studies, our method demonstrates the seamless transferability of segmented results into *Drishti*. This capability effectively fosters a new avenue for authentication and enhanced scrutiny of segmented data. By facilitating this interoperability, our approach underscores the potential for significant advancements in accuracy validation and collaborative research efforts across diverse scientific domains.

## 1. Introduction

Nowadays, 3D visualization and segmentation have become standard solutions for many different purposes [1–4] and one of the most valuable tools for research investigations, revealing hidden information behind the data [5–8]. For the last two decades, researchers in the 3D scientific visualization field have been experimenting with new ways to visualize medical and scientific data sets [3,9–18] more accurately. In the current workflow of the 3D scientific visualization process, 3D rendered results can be generated using combinations of image processing, segmentation and rendering techniques [18–20]. However, current 3D visualization software lacks interchangeability and contains natural barriers due to compatibility (e.g. format incompatible between different software), accessibility (e.g. manageability of data sharing is limited between collaborated parties) and affordability. The extended time to master each software and the complexity behind different algorithms or approaches are also a concern. These barriers weaken the achievability of scientific research’s transparency, replicability and reproducibility, damage the validation of communications between the collaboration of institutions, and restrict the authenticity assessment of a research finding, a proposed solution or a hypothesis.

While uploading surface mesh data of the segmented results of the study object enhances comprehension of the research, this approach still limits the ability of re-investigation by peers, as surface mesh data only contains external information [14,21–24]. A more efficient and resource-friendly way is to use the segmented volume data, which includes both internal and external information.

Digital Imaging and Communications in Medicine (DICOM) is well-known in medicine for being exchangeable between any two entities in biomedical imaging and analysis software [25]. Most mainstream 3D visualization software (e.g. Mimics, Avizo and VG Studio) can input and output DICOM. In medical science, segmented DICOM data from a scan (e.g. ultrasound or MRI) can be used to re-analyse segmented regions of interest using other software for diagnostics [26,27]. Thus, we developed volume exploration and presentation software *Drishti* v.2.7 to solve the current barriers in image processing and 3D visualization software by implementing an ability to include both single and multiple DICOM directories of segmented volumetric data from other 3D visualization software. To our knowledge, this method conquers current compatibility and interchangeability constraints among 3D visualization software.

## 2. Material and methods

### 2.1. Segmented volume data for revisualization

Segmented volume data of a 410-million-year-old fossil fish *Tungsenia paradoxa* (IVPP V10687). Initial segmentation was done in Mimics [28]. The segmented braincase of an extant living fish *Erofoichthys* (IVPP OV2715) was scanned at IVPP. Both data were output as multiple zipped DICOM files from Mimics 18.0 (option: Export-Masks).

Segmented volume data of a Miocene bird *Linxiavis* [29]. Initial segmentation was done in Avizo. These data were output as multiple DICOM directories from Avizo 9.0 (option: Export-DICOM).

Segmented volume data of an extant lizard *Varanus indicus* (AMNH R58389) was done in VG Studio. These data were output as single DICOM directories from VG Studio 3.3 (option: Filter-Export-volume). The CT scanning of *V. indicus* was completed at the Microscopy and Imaging Facilities at the American Museum of Natural History (AMNH), with permission from the Herpetology Department of the AMNH.

**Figure 1.**
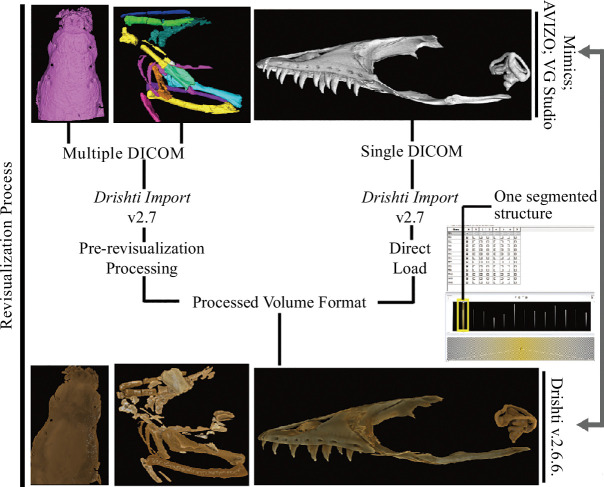
The general workflow for revisualization of segmented volume data in *Drishti* v.2.7.

The above four experiments were then imported into *Drishti* Import for processing (figure 1).

### 2.2. Procession of segmented volume data

We have implemented a new ability to import multiple DICOM volume datasets and collapse them into a single volume dataset. This was done to accommodate the segmentation results in numerous DICOM volumes, each consisting of a single segmented structure. Each segmented structure is assigned a voxel value, thereby merging all the segmented datasets into a single volumetric dataset to be visualized.

We use two illumination methods in our revisualization experiments, the global parameters, which include the properties of the overall scene (e.g. lights, shading, camera position and projection type) and object parameters, which include the properties of the segmented object (e.g. colour and opacity). Global parameters were used to operate the scene of a 3D visualization. Different parameters were used in revisualized segmented volume data (see electronic supplementary materials, figures S1–S3).

### 2.3. Image analysis

**Figure 2.**
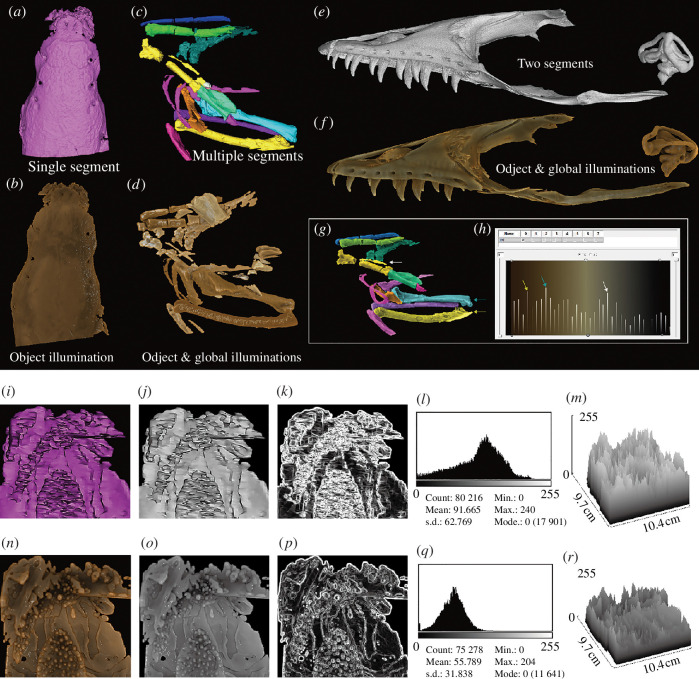
Segmented volume data from Mimics, Avizo and VG Studio and their corresponding revisualized segmented volume data from *Drishti*. (*a,b*) Dorsal view of the braincase of living fish *Erofoichthys* (IVPP OV2715) (*a,* Image obtained from Mimics for segmented volume; *b*, Image obtained from *Drishti* after revisualizing of segmented volume). (*c,d*) Lateral view of segmented fossil bird *Linxiavis* (IVPP V24116) (*c*, Image obtained from Avizo for segmented volume; *d,* Image obtained from *Drishti* after revisualizing segmented volume). (*e,f*) Lateral view of segmented lizard *V. indicus* (AMNH R58389) (*e*, Image obtained from VG Studio for segmented volume; *f*, Image obtained from *Drishti*, after revisualizing of segmented volume). (*g,h*) Demonstrates the capability of assigning individual colours to differentiate segmented structures, allowing each segmented layer of the initial segmentation to be shown separately in the 2D transfer function window with its voxel information. Three pairs of coloured arrows indicate three separate revisualized segmented structures (*g*) and their corresponding counterparts in the 2D transfer function window (*h*). (*i–r*) Comparison of the segmented braincase of living fish *Erofoichthys* (IVPP OV2715) in Mimics and *Drishti*. (*i–k*) Images obtained from Mimics. (*i*) Original image output from Mimics. (*j*) 8-Bit greyscale image of (*i*). (*k*) Edges in (*j*). (*l*) 2D histogram of (*j*). (*m*) The 3D surface plot of (*j*). (*n*–*r*) Images obtained from *Drishti* after revisualization. (*n*) Original image output from *Drishti* in the same orientation and scale of (*i*). (*o*) Revisualized result corresponds to (*j*). (*p*) Revisualized result corresponds to (*k*). (*q*) 2D histogram of (*o*). (*r*) The 3D surface plot of (*o*). The median optical density (OD) of (*j*) and (*o*) are 0.549 and 0.926, respectively, calculated using the calibrated curve displayed in electronic supplementary material, figure S1.

Images of the revisualized volume data were generated in the same orientation and scale in *Drishti* as in Mimics, Avizo and VG Studio. Images were analysed using ImageJ (https://imagej.nih.gov/ij/index.html) and ICY (http://icy.bioimageanalysis.org). Results were analysed using Microsoft Excel and displayed in §2 and electronic supplementary materials. ImageJ (https://imagej.nih.gov/ij/index.html) and ICY (http://icy.bioimageanalysis.org/) were used to analyse images to demonstrate their properties (figure 2 and see electronic supplementary materials, figures S1–S3). Images were input into ImageJ and ICY and then transformed from RGB to 8-bit greyscale images for analysis. 2D histogram of pixel intensity, 3D surface plot and edges on those images were generated per image. The 2D histogram shows the distribution of grey values in that image. The *x*-axis represents the possible grey values and the *y*-axis indicates the number of pixels found for each grey value. The 3D surface plot displays a 3D graph of the intensities of pixels in an 8-bit greyscale colour image. Edges were calculated using the Sobel Edge Detection algorithm [30]. The results of this analysis are displayed here (figure 2).

The median optical density (OD) of each selected image (electronic supplementary material, figure S4) was measured in ImageJ for further analysis and displayed in electronic supplementary material, figure S5. Calibration of OD (see electronic supplementary material, figure S7) was done using a Kodak No. 3 Calibrated Step Tablet scanned with an Epson Expression 1680 Professional scanner. The tablet has 21 steps with a density range of 0.05–3.05 OD (see electronic supplementary material, figure S7*a*). There is a weak correlation and positive linear relationship between images of the revisualized segmented volume using *Drishti* and images of the segmented volume in Mimics (see electronic supplementary material, figure S6).

## 3. Results

To test this new revisualization method, we use four segmented volumetric data from three mainstream 3D visualization software—Mimics, VG Studio and Avizo as case studies. The four segmented volume data have been saved as single (VG Studio) and multiple DICOM (Mimics and Avizo) directories, and subsequently imported into *Drishti* v.2.7 (or later version) to transform and convert into processed volume formats which *Drishti* can read and process. After proceeding to the processed volume format (i.e. pvl.nc), segmented volume data can be explored and rendered in *Drishti* (figures 1 and 2). We use 1D and 2D transfer functions with different illumination methods, such as applying different opacity and light volumes per layer or segmented structures to revisit the segmented volume data ( figure 2 ). Our approach also allows each segmented layer of the initial segmentation to be shown separately in the 2D transfer function window with its voxel information (figure 2). The 2D transfer function then maps the voxel information of each layer to optical properties before applying different illumination methods. Voxel intensity per layer can be read separately or together using the transfer function editor ( figure 2 ).

Revisualized segmented volume data can be exported in three standard formats using *Drishti Import*: RAW, greyscale 8-bit unsigned image stacks and ITK MetaImage format, which are considered common formats that all image processing and 3D visualization software can read and process. Revisualized segmented volume can also be merged with original volume data to enhance a region of interest or area, validating the precision and accuracy of segmented results. To achieve this, two volumes, the revisualized segmented volume and the original tomogram, need to be loaded together in *Drishti Render*, rendered then saved as an Extensible Markup Language data file (i.e. .xml). As demonstrated, this new revisualization method achieves the interchangeability between different image processing and 3D visualization software. This method generally works for any software that allows DICOM as an output format. During the revisualization process, 2D and 3D images, movies and other visuals can also be generated.

## 4. Conclusion

Our revisualization method illuminates detailed information on segmented volume data through different combinations of illumination algorithms and features. It uses mainstream image processing and 3D visualization software and maximizes the 3D visualized outcome of any segmented volume data while maintaining uniformity and interoperability between different software platforms and volumetric data formats. This revisualization method is an efficient way to break current restrictions and barriers due to compatibility, accessibility and affordability issues among different visualization software. In addition, it is straightforward enough to be widely used and maintains the capability of producing more intuitive visual representations, improving collaboration efficiency.

## Data Availability

Step-table and calibrated curve for optical density; one movie for demonstration of the suggested workflow of revisualization; code is available at [31]. All segmented datasets are available online at [32]
